# Methodological aspects of a GIS-based environmental health inspection program used in the Athens 2004 Olympic and Para Olympic Games

**DOI:** 10.1186/1471-2458-5-93

**Published:** 2005-09-02

**Authors:** Christos Hadjichristodoulou, Elpidoforos S Soteriades, Virginia Kolonia, Matthew E Falagas, Efstathios Pantelopoulos, Georgios Panagakos, Varvara Mouchtouri, Jeni Kremastinou

**Affiliations:** 1 Department of Hygiene and Epidemiology, Faculty of Medicine, University of Thessaly, Larissa, Greece; 2 National School of Public Health – Olympic Planning Unit (OPU), Athens, Greece

## Abstract

**Background:**

The use of geographical information system (GIS) technologies in public health surveillance is gradually gaining momentum around the world and many applications have already been reported in the literature. In this study, GIS technology was used to help county departments of Public Health to implement environmental health surveillance for the Athens 2004 Olympic and Para Olympic Games.

**Methods:**

In order to assess the workload in each Olympic county, 19 registry forms and 17 standardized inspection forms were developed to register and inspect environmental health items requiring inspection (Hotels, restaurants, swimming pools, water supply system etc), respectively. Furthermore, related databases were created using Epi Info 2002 and a geographical information system (GIS) were used to implement an integrated Environmental Health inspection program. The project was conducted in Athens by the Olympic Planning Unit (OPU) of the National School of Public Health, in close cooperation with the Ministry of Health and Social Solidarity and the corresponding departments of Public Health in all municipalities that were scheduled to host events during the Athens 2004 Olympic and Para Olympic games.

**Results:**

A total of 44,741 premises of environmental health interest were geocoded into GIS databases and several electronic maps were developed. Using such maps in association with specific criteria, we first identified the maximum workload required to execute environmental health inspections in all premises within the eleven Olympic County Departments of Public Health. Six different scenarios were created for each county, based on devised algorithms in order to design the most effective and realistic inspection program using the available inspectors from each municipality. Furthermore, GIS applications were used to organize the daily inspection program for the Olympic games, provide coloured displays of the inspection results and link those results with the public health surveillance of specific cases or outbreak investigation.

**Conclusion:**

Our computerised program exhibited significant efficiency in facilitating the prudent use of public health resources in implementing environmental health inspections in densely populated urban areas as well as in rural counties. Furthermore, the application of simple algorithms in integrating human and other resources provided tailored and cost-effective applications to different public health agencies.

## Background

The Athens 2004 summer Olympic and Para Olympic games, was the largest sport event ever held in Greece, and involved a large number of athletes and spectators gathered in the greater metropolitan area of Athens and four other Olympic Cities. Protecting the health of the athletes, officials, spectators and the population of Athens during the Olympic games, was one of the top priorities of the public health professionals and the Greek government itself. The Ministry of Health and Social Solidarity, as early as three years prior to the games had assigned the different tasks for public health preparation to the appropriate agencies. The National School of Public Health in Athens, in cooperation with the Greek Ministry of Health, put together a team of public health professionals in order to form the Olympic Planning Unit (OPU), which took the responsibility of coordinating the Environmental Health Inspection programs in the Olympic cities.

The idea of implementing a comprehensive environmental health inspection program for the Athens 2004 Olympic Games, using a geographical information system (GIS), was based on previous reports by epidemiologists and environmentalists regarding the spatio-temporal associations between environmental exposures and distribution of diseases in the population [1-4]. The importance of place in relation to population health has long been implicated in ancient as well as in modern epidemiology [5]. Using GIS software along with geocoding, researchers utilize powerful tools in exploring the relationship between geographic location and health [6]. Many environmental health studies examined the use of GIS in the field of geographical epidemiology by drawing up disease maps and conducting ecological analyses [7]. GIS technology has also been increasingly used in the mapping of exposures of certain environmental hazards, which might disproportionately affect human populations [8]. Maantay has described a GIS-based environmental equity study, conducted in the past decade, reviewing the spatial relationship between environmental pollution and health [9]. In addition, the value of GIS has been assessed for health care planning within research and practical applications [10], for planning of joint health and social care services [11], and for assessing areas with shortage of physicians [12].

Furthermore, specific capabilities of the GIS technology, which allow users to produce clear and accessible maps in association with geocoded data reports, constitute a powerful tool that were deemed extremely helpful to our main objective [13,14]. While GIS technology had also been used in the surveillance of communicable diseases (real time outbreak and disease surveillance – RODS), there was no previous report on the use of GIS in Environmental Health Inspection programs in previous Olympic Games [15].

In this report, we present the methodology used to develop a comprehensive environmental health inspection program for the Athens 2004 Olympic and Para Olympic Games, using GIS. In addition, we demonstrate several applications of the GIS databases in scheduling the environmental health inspections and allocating human resources according to the workload of specific geographic regions.

## Methods

The Olympic planning unit of the National School of Public Health initiated the public health preparations in the field of environmental health by identifying a number of public health risks in order to design and coordinate an effective environmental health inspection program to protect the health of both visitors and residents during the Olympic period. Food safety, drinking, and recreational water safety, as well as other items such as pest control, waste management (solid and liquid), legionella prevention (water supply systems, cooling towers, decorative fountains), public toilet sanitation and vessel sanitation were determined as the main items of public health interest and the most appropriate targets of the environmental health inspection program. According to experience from previous Olympic Games and other mass gatherings, the most frequent public health problems included food borne and/or waterborne outbreaks [15,16].

In order to design and coordinate an environmental health inspection program, the National School of Public Health – Olympic Planning Unit (NSPH – OPU), developed a close cooperation with a network of public health professionals from the office of environmental health at the Ministry of Health, the office of medical services from the Athens 2004 Olympic Games Organizing Committee and all county departments of public health responsible to oversee inspection programs in municipalities hosting Olympic events.

### Olympic county departments of public health

The vast majority of Olympic events were scheduled to take place in the metropolitan area of Athens, which included four sections of the municipality of Athens (central, east, west and south), the municipalities of East and West Attica, and the municipality of Piraeus with its main port. Additional counties hosting Olympic events included the metropolitan area of Thessalonica with its second largest port in the north of the country (the second largest city of Greece), the metropolitan area of Patra with its port, and two smaller cities; the city of Volos and the city of Iraklio. Environmental health inspectors from the seven County Departments of Public Health (CDPH) of the greater metropolitan area of Athens and the CDPH of the other four Olympic cities (Thessalonica, Patra, Volos, and Iraklio) were responsible for conducting the environmental health inspections prior to, and during the Olympic and Para Olympic Games.

### Workload assessment

To assess the workload of county departments of public health (CDPH) located in Olympic cities, 19 specific registry forms and 17 standardized inspection forms were created. Public health inspectors identified and registered all premises of public health interest in each Olympic CDPH, in advance, via on-site visits, using the registry forms. The premises targeted were the following: Olympic Venues, hotels, archaeological sites, cruise ships, camps, seacoasts, airports, marines, bottle-water plants, ice-producing plants, restaurants, other food premises, canteens, water supply systems for buildings and vessels, swimming pools, decorative fountains, cooling towers, waste management facilities, sewage treatment units, public toilets, and areas requiring pest control. Corresponding to the above-described forms, related databases were created using Epi Info 2002 in order to record basic information for the eligible inspection items and document the inspection results [17].

The exact address and postal code of each premise of environmental health interest and each archaeological and tourist area were used to develop GIS maps for each county. By employing the Arc view 3.2 software, all registered addresses were geocoded into specific databases [18]. The longitude and latitude coordinates for every geocoded address were assigned automatically from the digital map (called a street reference map), so that we would visualize the location of each specific place. Using the above maps, we estimated the total miles along with the mean inspection time required to inspect each registered premise, by taking into account the inspection time itself and the one-way transportation time to each premise. The transportation time was calculated using GIS simulated travel time on a particular street network. Pedestrian streets, one-way roads and specifically designated Olympic lanes and other restrictions employed during the Olympic games were taken into consideration for the calculation of the transportation time.

The following criteria were considered as constant parameters used to estimate the workload of each county (1) all inspections are performed by two inspectors (a pair). The assignment of inspectors in pairs was based on their experience in inspecting food premises or water sites in order to achieve complementary expertise; (2) the daily working time is estimated at 6 hours including transportation and inspection time; (3) the inspectors' transportation speed was estimated, using our own inspection data. Driving was estimated at about 10 kilometers (km) per hour (h), and walking on a pedestrian zone at about 4 km/h, and on stairways at about 2 km/h, respectively; and (4) the starting point of every daily route was considered to be each CDPH office. Using the above criteria, we calculated the total number of inspection hours needed for each CDPH. The number of working days, for every inspector, was estimated by dividing the total number of estimated inspection hours by six hours, which represented the daily working time for inspectors. In addition, we estimated the required person-time (in months) for the total number of inspections (each month was considered as 22 working days).

### Designing the environmental health inspection program

The Olympic environmental health inspection program had two main components for two time periods (the Pre-Olympic and the Olympic period). The first component referred to the inspections inside the Olympic Venues and the second component to the inspections outside the Olympic Venues. Therefore, the program was modified for each component and time period accordingly. Environmental health inspections inside the Olympic Venues were scheduled to be conducted strictly by certified inspectors. All environmental health inspections included on-site inspections and selective sampling for chemical and microbiological examinations. In order to achieve high quality and operational stability, the inspections were standardized and were performed based on standardized checklists and sampling forms. Seventeen standardized inspection forms were created using a negative scoring system [see additional files 1, 2]. All inspected premises were ranked in three categories (A, B or C) according to their score. Category A represented the highest score and therefore the best premises with respect to public health risks.

In order to cope with the enormous number of existing food premises (restaurants, bars, canteens) in the area of Athens, and devise the most realistic and cost-effective environmental health inspection program for the Olympic Games, a series of scenarios was developed, including a different number of food premises in each scenario, which depended on the available number of inspectors, cars and financial resources of each Olympic CDPH.

The first scenario included inspections of all premises in all eleven Olympic CDPHs. In this first scenario, a large number of inspectors would be required to work for a long period of time in order to cover the total number of inspections. For example, in the central section of Athens, inspecting all 8,358 premises would require five pairs of inspectors working for 24.9 months. Apparently this scenario was not realistic because it would impede counterchecks of several locations whenever needed. The second scenario estimated the time and personnel required for the inspection of hotels and Olympic Venues only. However, this would not be sufficient for protecting public health during the Olympic and Para Olympic Games. The third, fourth and fifth scenarios included the inspection of all premises in each Olympic county and the inspection of all food premises around Olympic Venues and Olympic hotels in buffers of 1,000, 500 and 300 meters, respectively. The above scenarios enabled us to conduct a sufficient number of inspections; however a big number of food premises were not included. Finally, the sixth scenario, which was recommended and selected for implementation, included the following: (1) inspection of all premises with the exception of food premises, in each Olympic CDPH; (2) inspection of all food premises around Olympic Venues and Olympic hotels in a buffer of 200 meters; (3) inspection of all food premises around touristy and archaeological areas in a buffer of 200 meters; and (4) inspection of 2% of the food premises, randomly selected, from the rest of each county.

### Partitioning of the Olympic counties

An algorithm was developed and utilized based on GIS technology in order to partition each Olympic CDPH in as many equal parts as the number of available pairs of inspectors. The term equal did not refer to the geographical area or the number of premises eligible for inspection. Rather the partitioning was based on the workload of each CDPH (required time for inspecting all identified locations of environmental health interest). Therefore, we organized the daily inspection program in such a way that the available inspectors in each county would perform the maximal possible number of inspections during the Pre-Olympic and Olympic period.

### Other GIS applications of the program

By using databases from Epi info 2002 and its GIS component, we were able to develop additional applications in order to implement the daily inspection program. For example, we were able to display the inspection results in color codes according to the categorical score received by each premise. Furthermore, we had the ability to link the inspection results to information regarding the surveillance of human cases of Legionnaire's disease.

## Results

### Olympic counties' workload

**Figure 1:**
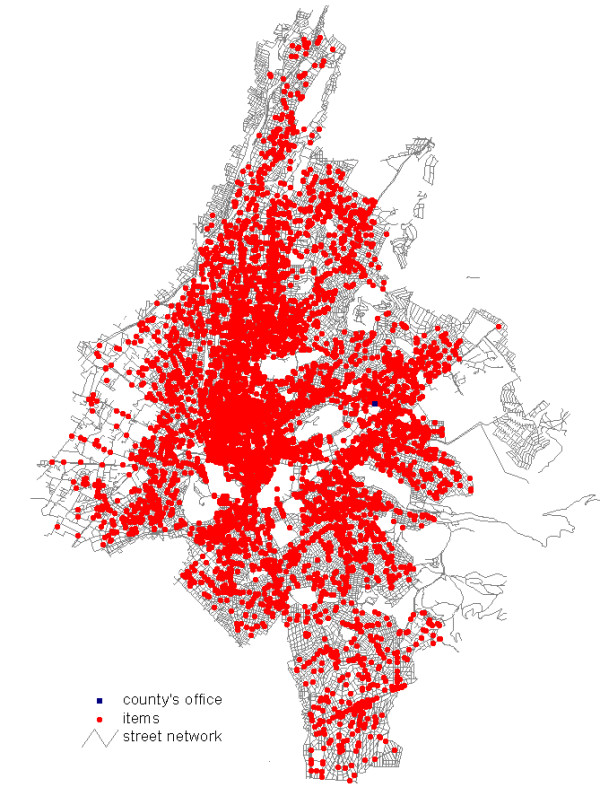
The total number of registered premises of environmental health interest represented by red dots (Olympic Venues, hotels, restaurants, camps, swimming pools, cooling towers etc.) in the central section of Athens, which were eligible for inspection.

A total of 44,741 premises of environmental health interest were registered using several reporting forms. As a result, 44,741 addresses were geocoded into GIS databases and developed into appropriate working maps. In Table 1 we present the total number of registered premises in each Olympic County Department of Public Health. In Figure 1 we provide an example of a GIS map produced, representing all premises of environmental health interest of the central section of Athens, which was the most important Olympic county since most of the Olympic events took place within its boundaries.

**Table 1:** Total number of registered premises of environmental health interest in each Olympic County Department of Public Health (CDPH) in Greece

**County Department of Public Health**	**Olympic Venues**	**Hotels**	**Food Premises**	**Other Premises**	**Total**
Central Athens	5	254	7,954	145	**8,358**
Eastern Athens	5	22	2,117	133	**2,277**
Southern Athens	3	53	2,467	125	**2,648**
Western Athens	1	5	2,570	108	**2,684**
Eastern Attica	6	80	1,961	100	**2,147**
Western Attica	1	15	1,463	57	**1,536**
Piraeus	3	258	5,356	224	**5,841**
Thessalonica	1	111	9,927	57	**10,096**
Patra	1	100	3,112	49	**3,262**
Volos	1	238	2,522	77	**2,838**
Iraklio	1	466	2,484	103	**3,054**
**Total**	**28**	**1602**	**41,933**	**1,178**	**44,741**

### Olympic inspection program

**Figure 2:**
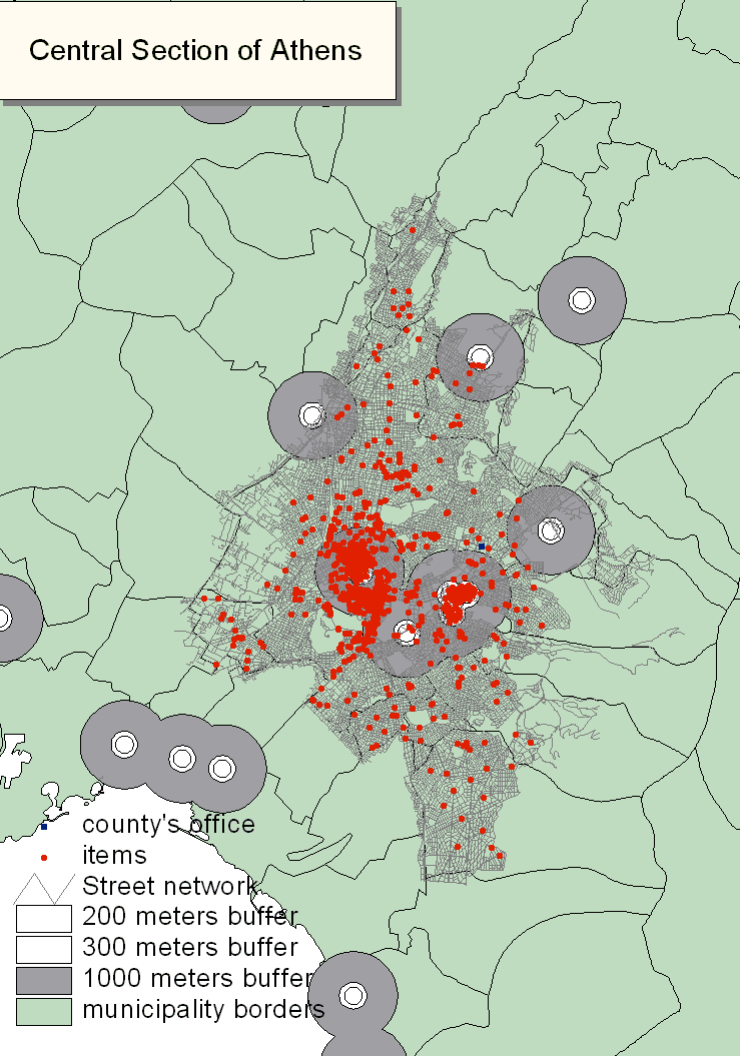
The total number of premises represented by small red dots (Olympic Venues, hotels, restaurants, camps, swimming pools, cooling towers etc.) requiring inspection in the central section of Athens (shaded in grey), as defined by the computerized program applying the sixth scenario (described in the text). Buffers with increasing size are displaying areas of public health interest around Olympic Venues and tourist and archaeological sites.

Based on the sixth scenario described in the methods section, we determined the exact number and type of premises that were to be inspected in each CDPH during the Pre-Olympic and Olympic period. For instance, the workload of the central section of Athens was estimated at 1,250 hours or 420 person-days for the inspection of 1,010 environmental health items. Therefore, the total inspection time for 5 pairs of inspectors was estimated at 4.7 months. In Table 2 we compare the application of two different scenarios; the first and the sixth scenario; for all Olympic CDPH and in Figure 2 we present a GIS image of the recommended scenario in the central section of Athens.

**Table 2:** Comparison of scenario 1 (inspection of all county premises) to scenario 6 (inspection of a selected number of premises) in each Olympic CDPH in Greece

	**Scenario 1**		**Scenario 6**	
	
**County Department of Public Health**	**Number of Premises for inspection**	**Required time (in months)**	**Number of Premises for inspection**	**Required time (in months)**
Central Athens	8358	24.9	1010	4.7
East Athens	2277	9.1	578	2.8
South Athens	2648	9.4	929	3.8
West Athens	2684	9.6	640	2.9
East Attica	2147	8.8	386	2.6
West Attica	1536	5.7	171	2.4
Piraeus	5841	17.9	1532	6
Thessalonica	10096	29.7	599	3.5
Patra	3262	11.4	344	3.6
Volos	2838	10.4	429	4.3
Iraklio	3054	11.9	704	4.9
**Total**	**44741**	**148.8**	**7322**	**41.5**

### Partitioning

**Figure 3:**
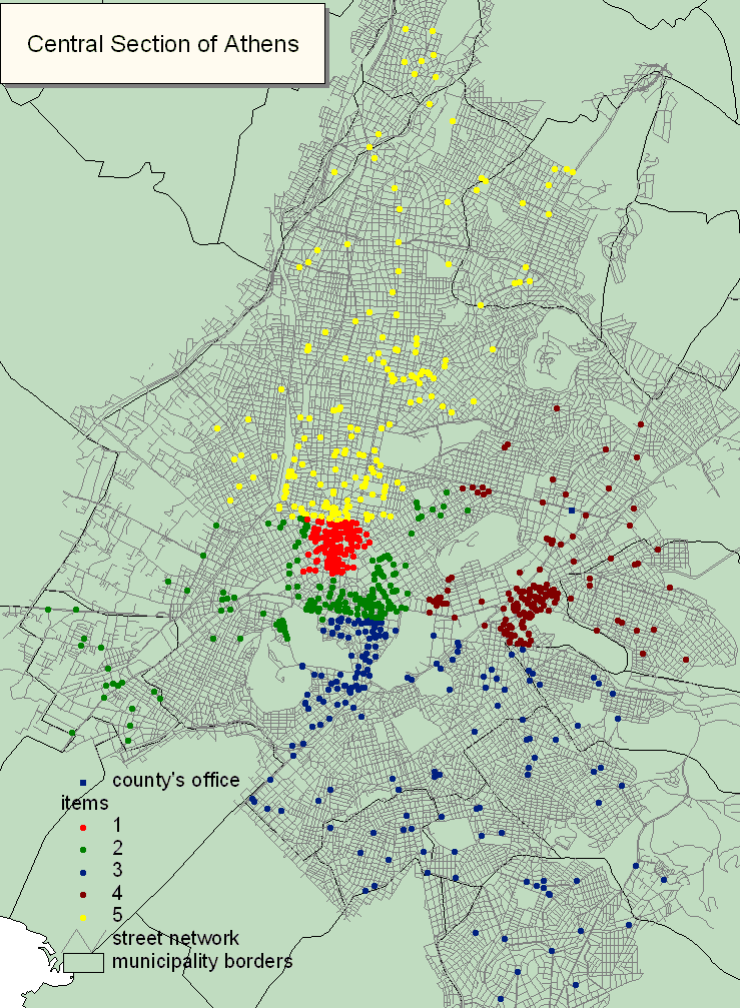
Partitioning of the central section of Athens in five parts with equal workload in terms of premises of environmental health interest requiring inspection based on the sixth scenario. Each part is displayed by a different color representing the workload of each pair of inspectors from the specific county.

The partitioning of every Olympic county was based on the available number of inspectors (pairs) employed in each county. For example, the central section of Athens, where five pairs of inspectors were employed, was partitioned in five parts with equal workload as it is shown in Figure 3.

### Other GIS applications

**Figure 4:**
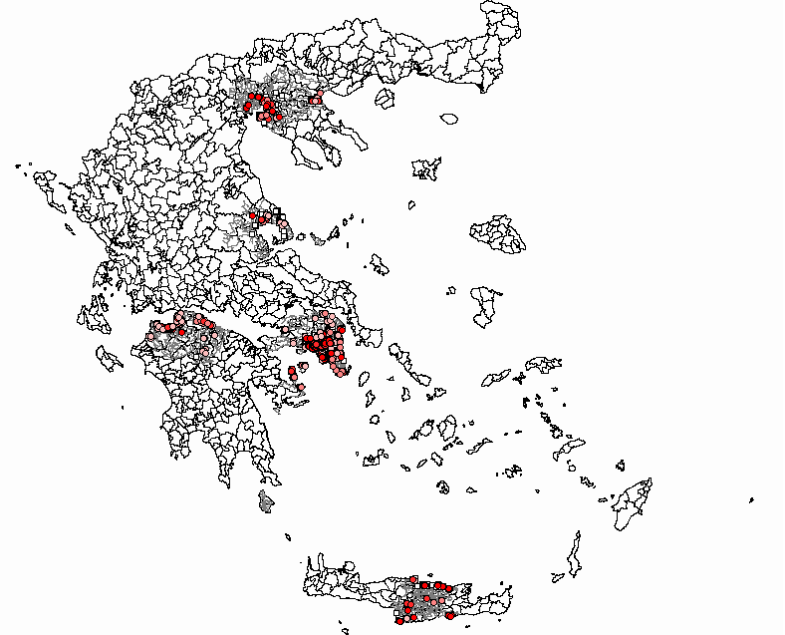
**(A)** Screenshot of Epi Map (part of US CDC Epi Info) [17] showing inspection results, on a zoomable GIS map, of all Olympic cities in Greece. The dark red dots and dark pink colored dots represent the premises, which received inspection score A and B, respectively (satisfactory results). The light pink colored dots represent the premises with the worst inspection score (score C – unsatisfactory results requiring counterchecks). **(B)** Screenshot of Epi Map (part of US CDC Epi Info) [17] showing inspection results, on a zoomable GIS map, of the central section of Athens. The dark red dots and dark pink colored dots represent the premises, which received inspection score A and B, respectively (satisfactory results). The light pink colored dots represent the premises with the worst inspection score (score C – unsatisfactory results requiring counterchecks). White dots represent environmental health items that were not inspected. Information on the inspection scores and laboratory results were available to be viewed on the map by selecting a specific environmental item as indicated by the window on the bottom left of the figure.

**Figure 5:**
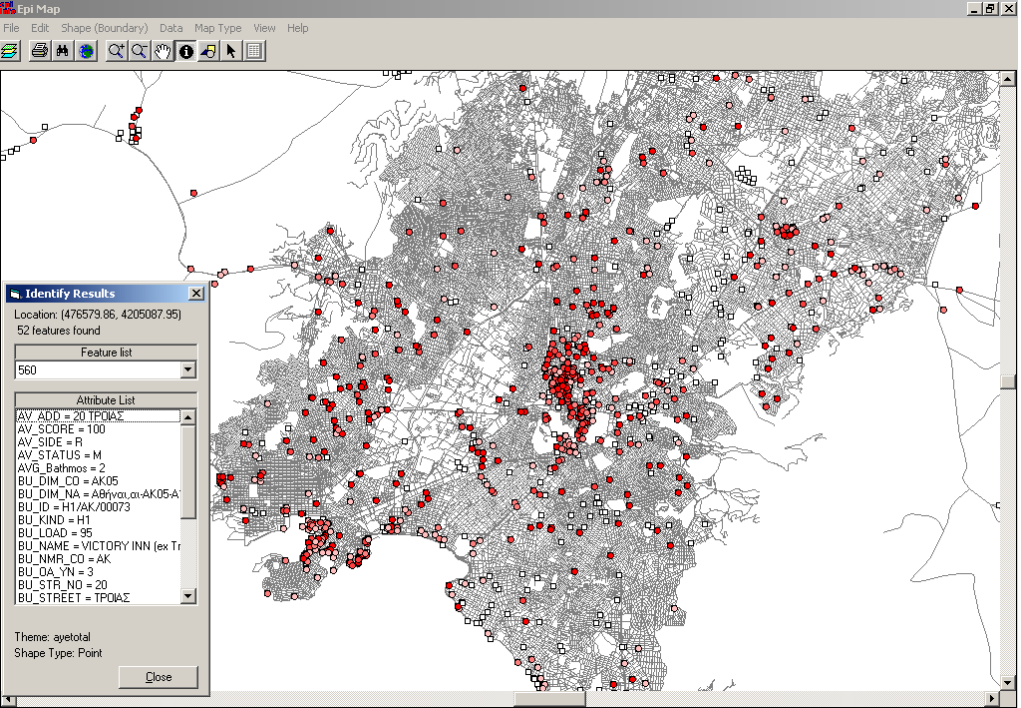
**(A)** Screenshot of Epi Map (part of US CDC Epi Info) [17] showing inspection results, on a zoomable GIS map, of the central section of Athens. The dark red dots and dark pink colored dots represent the premises, which received inspection score A and B, respectively (satisfactory results). The light pink colored dots represent the premises with the worst inspection score (score C – unsatisfactory results requiring counterchecks). White dots represent environmental health items that were not inspected. Information on the inspection scores and laboratory results were available to be viewed on the map by selecting a specific environmental item as indicated by the window on the bottom left of the figure.

The results of all environmental health inspections and all laboratory tests (microbiological examinations of the field samples obtained), were displayed on the electronic maps, in different colors according to the inspection score (score categories A, B or C), so that we would be able to automatically detect those premises that failed inspection and schedule counterchecks. For example, as we present in Figure 4 for the whole country and in figure 5 for the central section of Athens, 272 out of 781 premises of environmental health interest (34.83%) in the central section of Athens, inspected for the first time, received score A, 270 (34.57%) of the premises received score B and the remaining 239 (30.60%) received score C. All premises receiving score C had to undergo counterchecks within a month from the first inspection.

**Figure 6:**
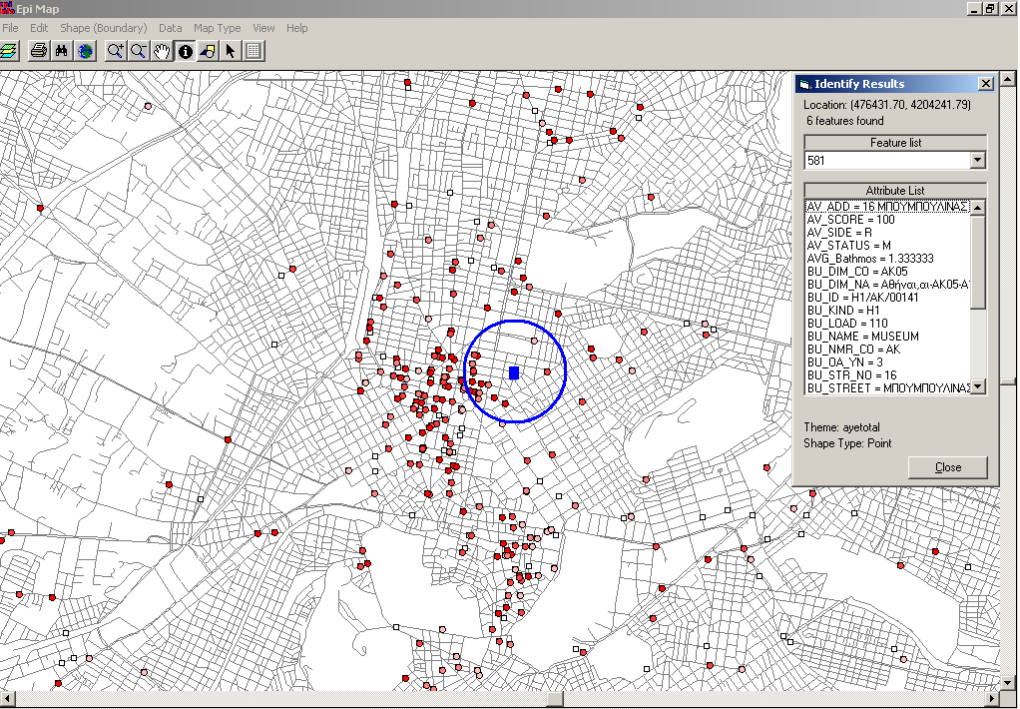
Screenshot of Epi Map (part of US CDC Epi Info) [17] showing all premises of environmental health interest (cooling towers, decorative fountains etc) in a buffer of 200 meters around a hypothetical case of legionnaires' disease in the central section of Athens depicted by a blue square (patients' residence). The dark red dots and dark pink colored dots represent the premises, which received inspection score A and B, respectively (satisfactory results). The light pink colored dots represent the premises with the worst inspection score (score C – unsatisfactory results). GIS map features included the ability to review, in real time, the inspection scores and laboratory results by selecting specific items within the buffer zone.

Another application was the correlation of potential cases of Legionnaires' disease in humans, with specific cooling towers or the water supply system of a specific building or even a decorative fountain in a specific region. For example, as it is presented in Figure 6, if a case of Legionnaires' disease would be detected in a person, who lived in a given address, all buildings of public health interest around the specific location, and included in a buffer of 200 meters would be evaluated automatically for previous inspection scores and laboratory results for legionella. Using this method the most possible source of infection could be identified.

## Discussion

To our knowledge, this is the first application of GIS technology that was used to plan, organize and implement the environmental health inspection program for the Olympic Games. The application of GIS technology along with data management and analysis, using the Epi info 2002, in the implementation of the Olympic environmental health inspection program, has been a valuable tool in selecting a realistic and cost-effective program that covered the most important premises of every Olympic county department of public health. Since visitors, spectators and athletes were expected to move, mostly, around hotels, Olympic Venues and highly tourist places, we believe that the recommended GIS-based inspection program represented one of the best approaches aiming to satisfy the demands of the Olympic Games in Athens while focusing on areas of sporting and entertaining events.

Moreover, a relatively low percentage (2%) of food premises outside the target geographical regions (buffers around Olympic venues and archaeological sites), were randomly selected for inspection in the pre-Olympic as well as during the Olympic period. In an effort to improve the beneficial effect of the inspections and diffuse the word around the Olympic cities about the environmental health inspection program, the results of the pre-Olympic period were publicized by the National Food Authority of the Ministry of Development.

Several useful applications of the GIS technology in Greece have been previously described such as in the development of operational systems to support decisions during large forest fire incidents [19]. In addition, it has been used for the implementation of an active surveillance program for brucellosis in a rural area of central Greece [20] and in the description of the profile of pollution of drinking water by nitrates, chloride and arsenic in Northern Greece [21]. We believe that the current application for the environmental health inspection program for the Athens 2004 Olympic and Para Olympic Games constituted a novel idea, which may be followed in future sporting events around the world.

We would like to acknowledge several limitations of our study. One possible source of error is the estimation of the workload of each county, which is based on criteria such as the inspectors' transportation speed, their daily working hours and the required inspection time for each item. For example, we are unable to predict, with reasonable accuracy, the speed of the inspectors and the duration of the inspections because both can be affected by unpredictable factors. In addition, two hours out of the six working hours of the inspectors were needed for completing the official reports used for each inspection and for other related subjects. Furthermore, the partition of the Olympic counties could be implemented taking into consideration primarily the distance and secondly the workload; however in that case, it would be very difficult to create a small number of partitions. Ideally, the results of the environmental health inspection program with GIS technology should be linked to the communicable disease surveillance for human cases. However, the above linkage was operational only for possible cases of legionella. A fully operational system for all communicable diseases was not in place due to limitations of human surveillance data (lack of geocoding).

## Conclusion

Despite the above restrictions, our program has proven its value whenever was tested during different events in the pre-Olympic and the Olympic period. Using our GIS-based inspection program, we have succeeded in covering all test events for the Olympic Games one year prior to the actual Olympic period with the least number of inspectors. Our program offered several advantages to public health authorities in Greece and may prove useful in designing, planning and implementing environmental health inspection programs in densely populated urban areas as well as in rural counties given its flexibility. In addition, the integration of human and other resources in simple algorithms provides tailored and cost-effective applications to each public health region. Furthermore, the GIS-based tools may support several other functions, described above, that allow inspectors to visualize the results on a colour scale, as well as associate them with other findings of public health importance such as data on outbreaks and or epidemics from public health surveillance. We believe that our method, as applied in the eleven Olympic counties in Greece, is relatively flexible and if accordingly modified, could be used by environmental health authorities in future Olympic Games and other mass gatherings around the world.

## Abbreviations

OPU – Olympic Planning Unit

GIS – Geographical Information System

CDPH – County department of public health

Km/h – Kilometers per hour

## Competing interests

The author(s) declare that they have no competing interests.

## Authors' contributions

CH, VK, EP, GP, and VM carried out the data collection. CH, ESS and VK drafted the manuscript. MEF, JK reviewed the manuscript and made significant comments. CH, VK, EP, GP, VM and JK participated in the design of the study. CH, EP and GP performed the statistical analysis. CH and JK conceived of the study, and along with EP and GP participated in its coordination. All authors read and approved the final manuscript.

## Pre-publication history

The pre-publication history for this paper can be accessed here:



## Supplementary Material

Additional file 1 Translated version of the standardized inspection form for restaurants Click here for file

Additional file 2 Translated version of the standardized inspection form for mobile canteens Click here for file

## References

[B1] Vine MF, Degnan D, Hanchette C (1997). Geographic information systems: their use in environmental epidemiologic research. Environmental Health Perspectives.

[B2] Moore DA, Carpenter TE (1999). Spatial analytical methods and geographic information systems: use in health research and epidemiology. Epidemiologic Reviews.

[B3] Bellander T, Berglind N, Gustavsson P, Jonson T, Nyberg F, Pershagen G, Jarup L (2001). Using geographic information systems to assess individual historical exposure to air pollution from traffic and house heating in Stockholm. Environmental Health Perspectives.

[B4] Miranda ML, Dolinoy DC, Overstreet MA (2002). Mapping for prevention: GIS models for directing childhood lead poisoning prevention programs. Environmental Health Perspectives.

[B5] Bonner MR, Han D, Nie J, Rogerson P, Vena JE, Freudenheim JL (2003). Positional accuracy of geocoded addresses in epidemiologic research. Epidemiology.

[B6] McElroy JA, Remington PL, Trentham-Dietz A, Robert SA, Newcomb PA (2003). Geocoding Addresses from a Large Population-based Study: Lessons Learned. Epidemiology.

[B7] Kistemann T, Dangendorf F, Schweikart J (2002). New perspectives on the use of Geographical Information Systems (GIS) in environmental health sciences. International Journal of Hygiene and Environmental Health.

[B8] Maantay J (2002). Mapping environmental injustices: pitfalls and potential of geographic information systems in assessing environmental health and equity. Environmental Health Perspectives.

[B9] Barnes S, Peck A (1994). Mapping the future of health care: GIS applications in health care analysis. Geographical Information Systems.

[B10] Foley R (2002). Assessing the applicability of GIS in a health and social care setting: planning services for informal carers in East Sussex, England. Social Science and Medicine.

[B11] Luo W (2004). Using a GIS-based floating catchment method to assess areas with shortage of physicians. Health and Place.

[B12] Boulos MN (2004). Towards evidence-based, GIS-driven national spatial health information infrastructure and surveillance services in the United Kingdom. Int J Health Geogr.

[B13] Boulos MN, Roudsari AV, Carson ER (2001). Health geomatics: an enabling suite of technologies in health and healthcare. J Biomed Inform.

[B14] Gesteland PH, Gardner RM, Tsui FC, Espino JU, Rolfs RT, James BC, Chapman WW, Moore AW, Wagner MM (2003). Automated syndromic surveillance for the 2002 Winter Olympics. J Am Med Inform Assoc.

[B15] Banwell K (2000). Environmental health preparation for the Sydney 2000 Olympic and Para Olympic Games. NSW Public Health Bulletin.

[B16] Hadjichristodoulou C, Mouchtouri V, Soteriades ES, Vaitsi V, Kolonia V, Vasilogiannacopoulos AP, Kremastinou J (2005). Mass gathering preparedness: the experience of the Athens 2004 Olympic and Para-Olympic Games. J Environ Health.

[B17] Epi Info. http://www.cdc.gov/epiinfo/.

[B18] Arc View 3.2. http://www.esri.com/software/arcgis/arcview.

[B19] Keramitsoglou I, Kiranoudis CT, Sarimvels H, Sifakis N A Multidisciplinary Decision Support System for Forest Fire Crisis Management DSS for forest fire crisis management. Environmental Management.

[B20] Hadjichristodoulou C, Soteriades E, Goutzianna G, Loukaidou M, Babalis T, Antoniou M, Delagramaticas J, Tselentis Y (1999). Surveillance of brucellosis in a rural area of Greece: application of the computerized mapping programme. European Journal of Epidemiology.

[B21] Fytianos K, Christophoridis C (2004). Nitrate, arsenic and chloride pollution of drinking water in Northern Greece. Elaboration by applying GIS. Environmental Monitoring and Assessment.

