# Supplementary material for: Methodological aspects of a GIS-based environmental health inspection program used in the Athens 2004 Olympic and Para Olympic Games
Source: BMC Public Health. 2005 Sep 2;5:93. doi: 10.1186/1471-2458-5-93 (PMC1232856; doi:10.1186/1471-2458-5-93)
Supplement: Additional file 1 — Translated version of the standardized inspection form for restaurants [file 1471-2458-5-93-S1.doc]

**INSPECTION REPORT FOR MOBILE CANTEEN**

Name: ………………………………. Code: __ __ __ __ __ __ __ __

# Date: ………/………/………… Inspection began at (time): ___ : ___

|  | INSPECTION POINTS | YES 🗸 | NO🞪 | NOTES |
| --- | --- | --- | --- | --- |
| **GENERAL POINTS** | | | | |
| **1**** | **A valid permit is held** |  |  |  |
| **2*** | **The location of the canteen is in good condition, far from unhealthy spots and the environment is kept clean** |  | **-3** |  |
| 3 | Walls constructed with solid material, smooth, waterproof, in good condition |  | -1 |  |
| **4*** | **No chairs and tables provided outside the canteen** |  | **-3** |  |
| FOOD | | | | |
| 5 | The food provided is according to the valid permit |  | -1 |  |
| **6*** | **Food and drinks are in good condition** |  | **-3** |  |
| **7*** | Food is properly transported, stored, prepared, displayed, and served |  | **-3** |  |
| **8*** | Good hygienic practices are applied during preparation and food service |  | **-3** |  |
| **EQUIPMENT – REFRIGERATORS** | | | | |
| 9 | Proper storage of clean equipment and utensils |  | -1 |  |
| **10*** | Sensitive food items are properly maintained in refrigerators |  | **-3** |  |
| **11*** | Ice cream storage and display meets temperature requirements (at least -10^0^ C) |  | **-3** |  |
| **12*** | Hot items are stored and served at temperature higher than 60^ο^C |  | **-3** |  |
| **13*** | Cold items are stored and served at temperature not exceeding 7^ο^C |  | **-3** |  |
| **14*** | Surfaces in contact with food are maintained clean |  | **-3** |  |
| WATER – WASTE – electric power | | | | |
| **15*** | **Water is provided from the water supply system or a water tank is well-maintained** |  | **-3** |  |
| 16 | Water heater is available with adequately hot running water |  | -1 |  |
| 17 | **Liquid waste disposed of properly** |  | -1 |  |
| 18 | Electric power continuously supplied |  | -1 |  |
| **solid waste disposal** | | | | |
| 19 | Solid waste containers with covers and automatic opening system are available and well maintained |  | -1 |  |
| **PEST MANAGEMENT** | | | | |
| 20 | Effective pest control is in place |  | -2 |  |
| Warewashing facilities | | | | |
| 21 | Existing facilities for cleaning and dis-infection of **dishes and utensils** are well maintained and functioning |  | -1 |  |
| 22 | Dishes and utensils properly cleaned according to health Instructions |  | -1 |  |
| lighting – ventilation | | | | |
| 23 | Adequate ventilation |  | -1 |  |
| 24 | Adequate lighting |  | -1 |  |

* Critical control point

** If No, the result is Unsatisfactory independently of the score

25. Result: Α …….. □ Satisfactory – (Total negative score: up to -4, no critical control point)

Β …….. □ Relatively satisfactory – (Total negative score: -5 to -8, or a critical control point)

Γ ......... □ Unsatisfactory – (Total negative score: more than -9)

Comments: ……………………………………………………………………….……………………………….

……………………………………………………………………………………………………………………………

Inspection ended at (time):___ : ___ Duration of inspection: ……………………….

Inspector:………………………………………………………………… Signature:……………………….
