# Supplementary material for: Methodological aspects of a GIS-based environmental health inspection program used in the Athens 2004 Olympic and Para Olympic Games
Source: BMC Public Health. 2005 Sep 2;5:93. doi: 10.1186/1471-2458-5-93 (PMC1232856; doi:10.1186/1471-2458-5-93)
Supplement: Additional file 2 — Translated version of the standardized inspection form for mobile canteens [file 1471-2458-5-93-S2.doc]

**INSPECTION REPORT FOR RESTAURANT**

Name: ………………………………. Code: __ __ __ __ __ __ __ __

# Date: ………/………/………… Inspection began at (time): ___ : ___

|  | INSPECTION POINTS | YES 🗸 | NO🞪 | NOTES |
| --- | --- | --- | --- | --- |
| **1**ΣΤΙΑΤΟΡΙΑ Κ.Τ.Λ. - Κ1ΝΤΟΣ ΟΝΤΟ000000000000000000000000000000000000000000000000000000000000000000000000000000000000000000000000** | **A valid permit is held** |  |  |  |
| **FOOD** | | | | |
| **2**** | **Food is in good condition according to food and drinks code** |  |  |  |
| **FOOD PROTECTION** | | | | |
| **3*** | **Potentially hazardous food items meet temperature requirements during transportation, storage, preparation, display and service** |  | **-3** |  |
| **4*** | **Food prepared at proper cooking temperatures / times** |  | **-3** |  |
| **5*** | **Hot food items are thawed at 5°C within four (4) hours** |  | **-3** |  |
| **6*** | **Potentially hazardous food items reheated so that the food core achieves a temperature of at least 75°C** |  | **-3** |  |
| **7*** | **Facilities provided to maintain potentially hazardous food at proper temperature (hot or cold) according to handling instructions** |  | **-3** |  |
| 8 | Food temperature measurement devises provided and properly located; Records are kept |  | -1 |  |
| 9 | Potentially hazardous food properly thawed |  | -1 |  |
| 10 | Food is generally protected during transportation, storage, preparation, display and service |  | -2 |  |
| **11*** | **Protection from cross-contamination between raw, ready to eat and cooked food. Food not re-served** |  | **-3** |  |
| 12 | Employees are prevented from cross-contaminating ready-to-eat food with bare hands by proper use of suitable utensils |  | -2 |  |
| 13 | Proper storage of utensils and other facilities (e.g. ice storage boxes etc.) |  | -1 |  |
| **PERSONNEL** | | | | |
| **14*** | **Food handlers are free of infections and other communicable diseases** |  | **-3** |  |
| **15*** | **Workers follow good personal hygienic practices (clothing, hair, gloves etc.)** |  | **-3** |  |
| 16 | **Food handlers are trained in food safety and health law** |  | -1 |  |
| **EQUIPMENT** | | | | |
| 17 | Food-contact surfaces properly designed, constructed and maintained |  | -2 |  |
| 18 | Surfaces not in contact with food are properly designed, constructed and maintained |  | -1 |  |
| **19*** | **Food-contact surfaces of equipment and utensils are clean;** |  | **-3** |  |
| 20 | Surfaces not in contact with food are clean |  | -1 |  |
| 21 | Single-use articles are properly used and not re-used |  | -1 |  |
| 22 | Ice-producing machine is clean and well maintained |  | -2 |  |
| **Cleanliness** | | | | |
| 23 | Adequate ware washing facilities; proper temperatures, sanitizers, and detergents are used |  | -2 |  |
| 24 | Equipment and utensils shall be air-dried or adequately drained before contact with food |  | -1 |  |
| **Water supply** | | | | |
| **25*** | **Water intended for human consumption** |  | **-3** |  |
| **26*** | **Safe water supply distribution system (function, maintenance)** |  | **-3** |  |
| **LIQUID WASTE** | | | | |
| **27*** | **Liquid waste disposed of properly** |  | **-3** |  |
| **TOILET AND HANDWASHING FACILITIES** | | | | |
| **28*** | **Adequate facilities, clean, and well equipped** |  | **-3** |  |
| **personnel TOILET AND HANDWASHING FACILITIES** | | | | |
| 29 | Adequate facilities, clean, equipped, and easily accessible |  | -2 |  |
| **solid waste disposal** | | | | |
| 30 | Solid waste containers are covered, of adequate number and pest proof. Solid waste disposal is frequently collected |  | -1 |  |
| 31 | Temporary solid waste disposal storage room is clean |  | -1 |  |
| **pest control** | | | | |
| **32*** | **Absence of insects, rodents, birds and other pests** |  | **-3** |  |
| 33 | Facility access / openings are protected. A pest management program exists |  | -1 |  |
| **floor - walls - Ceiling** | | | | |
| 34 | Construction, maintenance and cleanliness |  | -1 |  |
| **lighting - ventilation** | | | | |
| 35 | Adequate lighting. Adequate ventilation system is clean |  | -1 |  |
| **other services** | | | | |
| **36*** | **Toxic items stored, labeled and used properly** |  | **-3** |  |
| 37 | Clean, proper stored linen |  | -1 |  |
| **food transportation vehicles** | | | | |
| 38 | Proper design, construction, maintenance and cleanliness |  | -1 |  |
| **39*** | **Food contact surfaces are clean** |  | **-3** |  |

* Critical control point

** If No, the result is Unsatisfactory independently of the score

40. Result: Α …….. □ Satisfactory – (Total negative score: up to -7, no critical control point)

Β …….. □ Relatively satisfactory – (Total negative score: -8 to -14, or a critical control point)

Γ ......... □ Unsatisfactory – (Total negative score: more than -15)

Comments: ……………………………………………………………………….……………………………….

……………………………………………………………………………………………………………………………

Inspection ended at (time):___ : ___ Duration of inspection: ……………………….

Inspector:…………………………………………………………….Signature:……………………….
